# Identification CCL2,CXCR2,S100A9 of the immune-related gene markers and immune infiltration characteristics of inflammatory bowel disease and heart failure via bioinformatics analysis and machine learning

**DOI:** 10.3389/fcvm.2023.1268675

**Published:** 2023-11-16

**Authors:** Xu Luo, Rui Wang, Xin Zhang, Xin Wen, Siwei Deng, Wen Xie

**Affiliations:** ^1^ College of Clinical Medicine, Chengdu University of Traditional Chinese Medicine, Chengdu, China; ^2^ Department of Cardiology, Hospital of Chengdu University of Traditional Chinese Medicine, Chengdu, China

**Keywords:** bioinformatics, machine learning, heart failure, inflammatory bowel, biomarkers

## Abstract

**Background:**

Recently, heart failure (HF) and inflammatory bowel disease (IBD) have been considered to be related diseases with increasing incidence rates; both diseases are related to immunity. This study aims to analyze and identify immune-related gene (IRG) markers of HF and IBD through bioinformatics and machine learning (ML) methods and to explore their immune infiltration characteristics.

**Methods:**

This study used gene expressiondata (GSE120895, GSE21610, GSE4183) from the Gene Expression Omnibus (GEO) database to screen differentially expressed genes (DEGs) and compare them with IRGs from the ImmPort database to obtain differentially expressed immune-related genes (DIRGs). Functional enrichment analysis of IRGs was performed using Gene Ontology (GO) and Kyoto Encyclopedia of Genes and Genomes (KEGG). Subsequently, three machine models and protein–protein interactions (PPIs) were established to identify diagnostic biomarkers. The receiver operating characteristic (ROC) curves were applied to evaluate the diagnostic value of the candidate biomarkersin the validation set (GSE1145, GSE36807) and obtain their correlations with immune cells through the Spearman algorithm. Finally, the CIBERSORT algorithm was used to evaluate the immune cell infiltration of the two diseases.

**Results:**

Thirty-four DIRGs were screened and GO and KEGG analysis results showed that these genes are mainly related to inflammatory and immune responses. CCL2, CXCR2 and S100A9 were identified as biomarkers.The immune correlation results indicated in both diseases that CCL2 is positively correlated with mast cell activation, CXCR2 is positively correlated with neutrophils and S100A9 is positively correlated with neutrophils and mast cell activation. Analysis of immune characteristics showed that macrophages M2, macrophages M0 and neutrophils were present in both diseases.

**Conclusions:**

CCL2, CXCR2 and S100A9 are promising biomarkers that will become potential immunogenetic biomarkers for diagnosing comorbidities of HF and IBD. macrophages M2, macrophages M0, neutrophil-mediated inflammation and immune regulation play important roles in the development of HF and IBD and may become diagnostic and therapeutic targets.

## 1. Introduction

Heart failure (HF) is defined as any structural and/or functional failure of cardiac ejection, leading to complex clinical syndromes with typical symptoms and clinical signs (1), and it is the terminal stage of various cardiovascular diseases. HF has a high incidence rate and high mortality, and it leads to poor function and quality of life and high cost. HF affects over 64 million people worldwide (2). In view of the great burden of chronic HF to society, we need a deeper understanding of its pathophysiological mechanism, not only in terms of mortality but also in terms of the incidence rate related to repeated and long-term hospitalization, which merits accelerated research (3). The occurrence of HF is associated with cardiovascular aging (4), and risk factors include old age, hypertension, diabetes, dyslipidemia, obesity (5), volume overload, fluid congestion, hereditary cardiomyopathy, etc (6). Based on the ongoing exploration of the pathophysiological mechanisms of HF and the publication of clinical evidence-based research, inflammation and immunity, including autoimmune and infection-mediated mechanisms, are now considered other possible mechanisms of HF (7–9).

Inflammatory bowel disease (IBD) is a common and complex group of autoimmune diseases (10), including ulcerative colitis and Crohn’s disease which are chronic diseases of the gastrointestinal tract (11). In recent years, the incidence rate and prevalence of IBD have risen sharply in developed and developing countries (12), resulting in a significant global disease burden (13). The etiology and pathogenesis of IBD are still uncertain, and it may be related to genetic factors, environmental factors, and intestinal barrier dysfunction, as well as ecological imbalance of the gut microbiota and exacerbation of innate and adaptive immune functions (14, 15). IBD is a multisystem disease that primarily affects the gastrointestinal, musculoskeletal, eye, and skin systems, as well as the cardiovascular system (16).

The most recent research shows that IBD is related to cardiovascular disease, and HF events in IBD patients affect the disease process and prognosis (17). Patients with chronic inflammatory diseases (including IBD) are more likely to suffer from atherosclerotic cardiovascular disease, HF and atrial fibrillation (18). The interaction between IBD and HF is complex, and pathological and physiological changes mediated by immune factors are common features between the two diseases. Both diseases are related to the interaction and imbalance of immune cells (19, 20). Immune cells play a crucial role in the occurrence and development of both diseases. Therefore, it is necessary to identify common immune biomarkers for both diseases, evaluate immune cell infiltration in both diseases and determine changes in immune cell composition to elucidate the molecular mechanisms of HF and IBD development and develop new immunotherapy targets.

Bioinformatics is developing a bridge between computer science and medicine, and machine learning (ML) is a field of computer science that continuously improves the performance of learning tasks by exploring patterns in data and applying self-improvement. It involves the use of computers to simulate human learning (21). The application of bioinformatics and ML methods in the medical field can better help us understand the pathophysiological mechanisms of diseases, screen for disease-specific biomarkers and gain a deeper understanding of diseases. Therefore, this study uses a combination of bioinformatics and ML methods, along with the construction of PPI networks, to screen for immune-related gene markers common to both diseases, analyze the immune cell infiltration of HF and IBD and explore the immune-related mechanisms and targets of HF and IBD. The results may provide a new approach for the diagnosis and treatment of HF and IBD.

## 2. Materials and methods

### 2.1. Data downloading and processing

**Figure 1:**
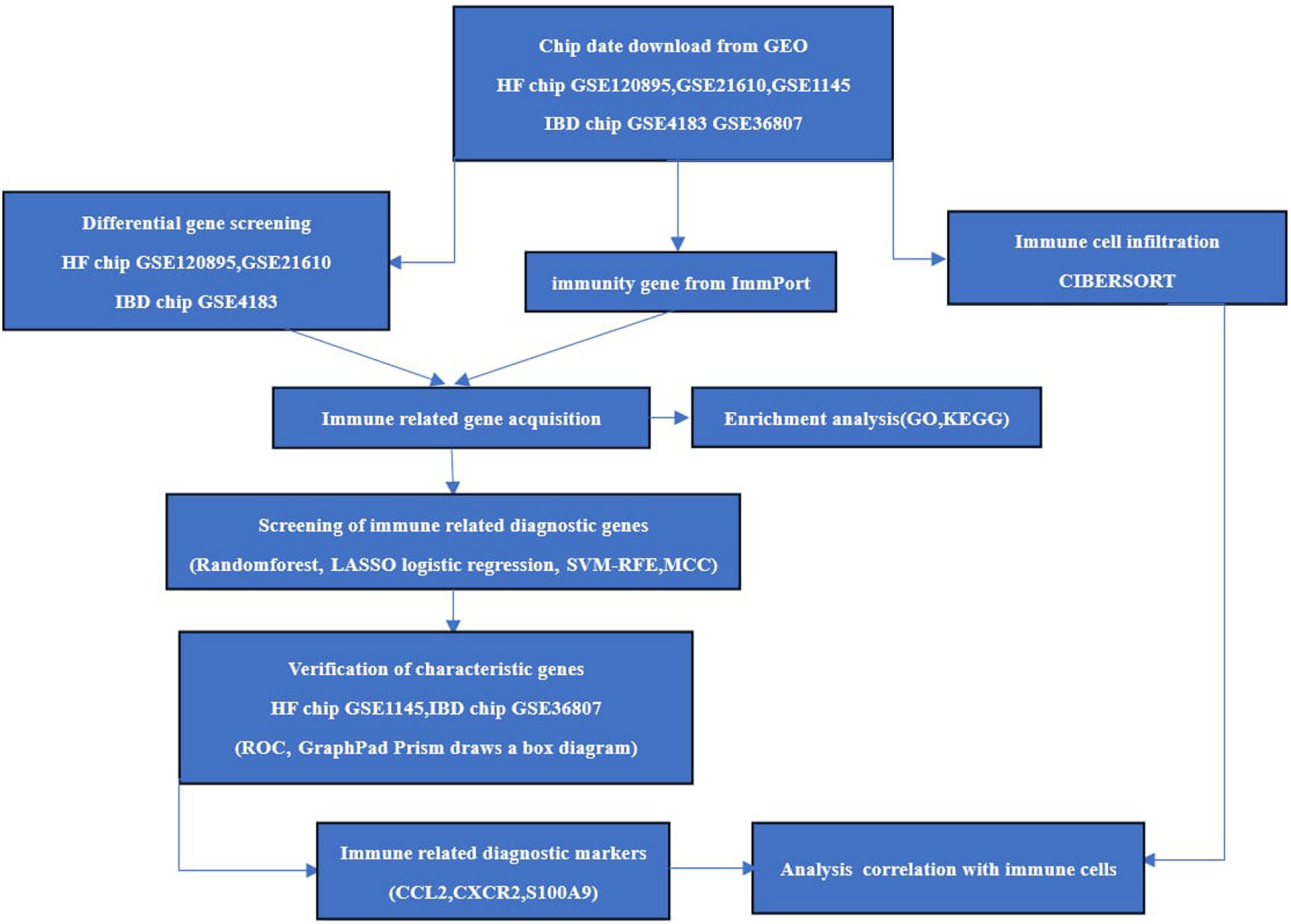
The study procedure.

The chips GSE120895 and GSE21610 for HF and GSE4183 for IBD were downloaded as experimental sets, and the HF chip GSE1145 and IBD GSE36807 datasets were downloaded as validation sets from the Gene Expression Omnibus (GEO) database (http://www.ncbi.nlm.nih.gov/geo/). Immune gene data were downloaded through the ImmPort database (https://www.immport.org/). R 4.3.0 software was applied to screen the differentially expressed genes (DEGs) of HF samples and normal samples on chips GSE120895 and GSE21610 and enteric disease samples and normal samples on GSE4183. The screening conditions were Abs log_2_Fold Change >1 and correction *P* < 0.05. The overlapping DEGs of the two diseases were introduced into DAVID (https://david.ncifcrf.gov) for Gene Ontology(GO) analysis and Kyoto Encyclopedia of Genes and Genomes (KEGG) pathway analysis. The specific process is shown in Figure 1.

### 2.2. Immune-related gene acquisition and functional correlation analysis

After clarifying that the overlapping genes of the two diseases were closely related to inflammation and immunity, the DEGs obtained from the HF and IBD chips and immune gene were intersectedto obtain immune-related genes for the two diseases. GO analysis and KEGG pathway analysis of immune-related genes were carried out to explore the biological functions and signaling pathways involved in immune-related genes.

### 2.3. Screening of diagnostic markers

Four methods are used for feature marker selection, including LASSO regression, random forest (RF), SVM-RFE and the MCC algorithm of the cytoHubba plug-in. The LASSO regression model is a multigene-based classifier (22) implemented through R’s glmnet software package, which is a high-dimensional variable regression analysis algorithm based on linear regression. The RF algorithm is an Ensemble learning algorithm based on decision trees. Each decision tree is randomly extracted from different samples and features, and then the results of these decision trees obtain the final prediction results by voting (23). The RF of R was used to sequence the immune-related genes of IBD and HF. By cross-validation with ten times the standard deviation of error t, the feature with the smallest error is selected, and the selected genes are identified as feature genes. The R package e1071 was used to construct the SVM classifier. Through tenfold cross validation, the SVM RFE algorithm was used in feature selection (24), and STRING 11.5 (https://cn.string-db.org/) was used to plot protein‒protein interaction (PPI) networks. Then, the MCC algorithm of the cytoHubba plugin was used. Finally, overlapping genes were selected from the above four classification models for further analysis.

### 2.4. Verification of diagnostic markers

The GSE1145 dataset of HF and GSE36807 dataset of IBD were used as the validation set. The diagnostic validity of the selected characteristic genes was evaluated with the ROCR package of R and MedCalc software using receiver operating characteristic(ROC) curves. The ROC curve and the area under the curve (AUC) of the subjects were used to evaluate the diagnostic efficacy. Statistical analysis was conducted using GraphPad Prism 9.5.1 software. Nonpaired sample t tests were performed on the expression levels of positive and normal samples, with a difference of *P* < 0.05 being statistically significant. The results were visualized in the form of box plots. Finally, the differences in the validation set were statistically significant; and, combined with the key genes of the ROC curve as the screening results of this study, it is believed that these may be immune-related diagnostic genes for HF and IBD.

### 2.5. Evaluation and correlation analysis of infiltration-related immune cells

The HF chip datasets GSE21610 and GSE120895, IBD chip GSE4183, and CIBERSORT’s LM22 immune cell dataset were used to study immune cell infiltration. This study used the CIBERSORT program of R to obtain immune cell infiltration data based on a cutoff of *P* < 0.05. Then, R’s ggplot2 package was used to draw a box plot of immune cell infiltration comparing positive and normal samples of the two diseases to compare the differences in immune cells between healthy individuals and patients. The Spearman relationship between immune-related biomarkers and 22 kinds of infiltrating immune cell-related heatmaps was drawn by using the “ggcorrplot” software package.

## 3. Result

### 3.1. Chip data acquisition

**Figure 2:**
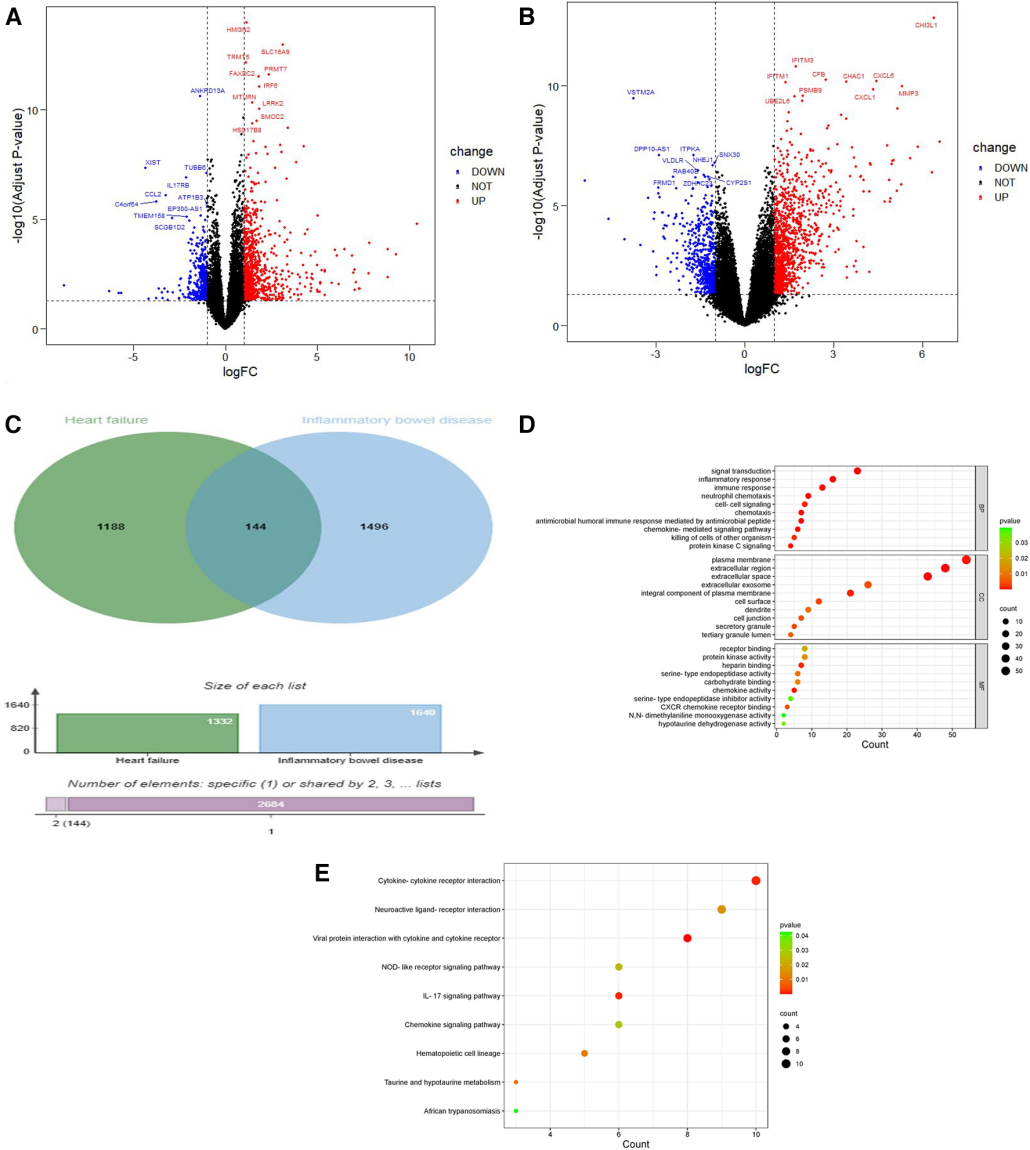
Volcano diagram and cross gene KEGG and GO diagram. (**A**) Volcano plot of HF. Blue dots represent downregulated genes, black dots represent nonsignificant genes, and red dots represent upregulated genes. (**B**) Volcano plot of IBD. Blue dots represent downregulated genes, black dots represent nonsignificant genes, and red dots represent upregulated genes. (**C**) Venn diagram of the overlap of HF DEGs and identical IBD DEGs that can be eliminated. (**D**) GO analysis of the functional enrichment of overlapping genes of HF and IBD. The dot size reflects the number of enriched genes, and the color indicates the significance of enrichment. (**E**) KEGG pathways of overlapping genes of HF and IBD. The dot size reflects the number of enriched genes, and the color indicates the significance of enrichment.

The experimental set of HF chip GSE21610 includes 60 HF samples and 8 normal samples; GSE120895 includes 47 HF samples of dilated cardiomyopathy and 8 normal samples. The IBD chip GSE4183 includes 15 IBD samples and 8 normal samples. After merging the HF chips GSE120895 and GSE21610 through R4.3.0 and removing batch effects, a total of 1,332 DEGs were obtained through comparative analysis with normal samples, including 924 upregulated genes and 408 downregulated genes compared to controls (Figure 2A). A total of 1,640 DEGs were obtained from IBD, including 1,059 upregulated genes and 581 downregulated genes compared to controls (Figure 2B). A total of 2,498 immune genes were obtained from the ImmPort databaseA total of 144 overlapping genes were obtained from the comparison of DEGs from HF and IBD (Figure 2C), and the overlapping genes were processed into GO and KEGG. The GO analysis (Figure 2D) showed that the overlapping genes were mainly enriched in the immune response, inflammatory response, chemokine activity, signal transduction, etc. Consistent with the results of GO analysis, KEGG analysis (Figure 2E) was significantly enriched in cytokine‒cytokine receptor interaction and signal transduction-related pathways. In addition, the chemokine signaling pathway and tumor necrosis factor signaling pathway were closely related to both diseases, indicating that both diseases are related to inflammation and immunity.

### 3.2. Acquisition of immune-related genes and GO and KEGG analysis

**Figure 3:**
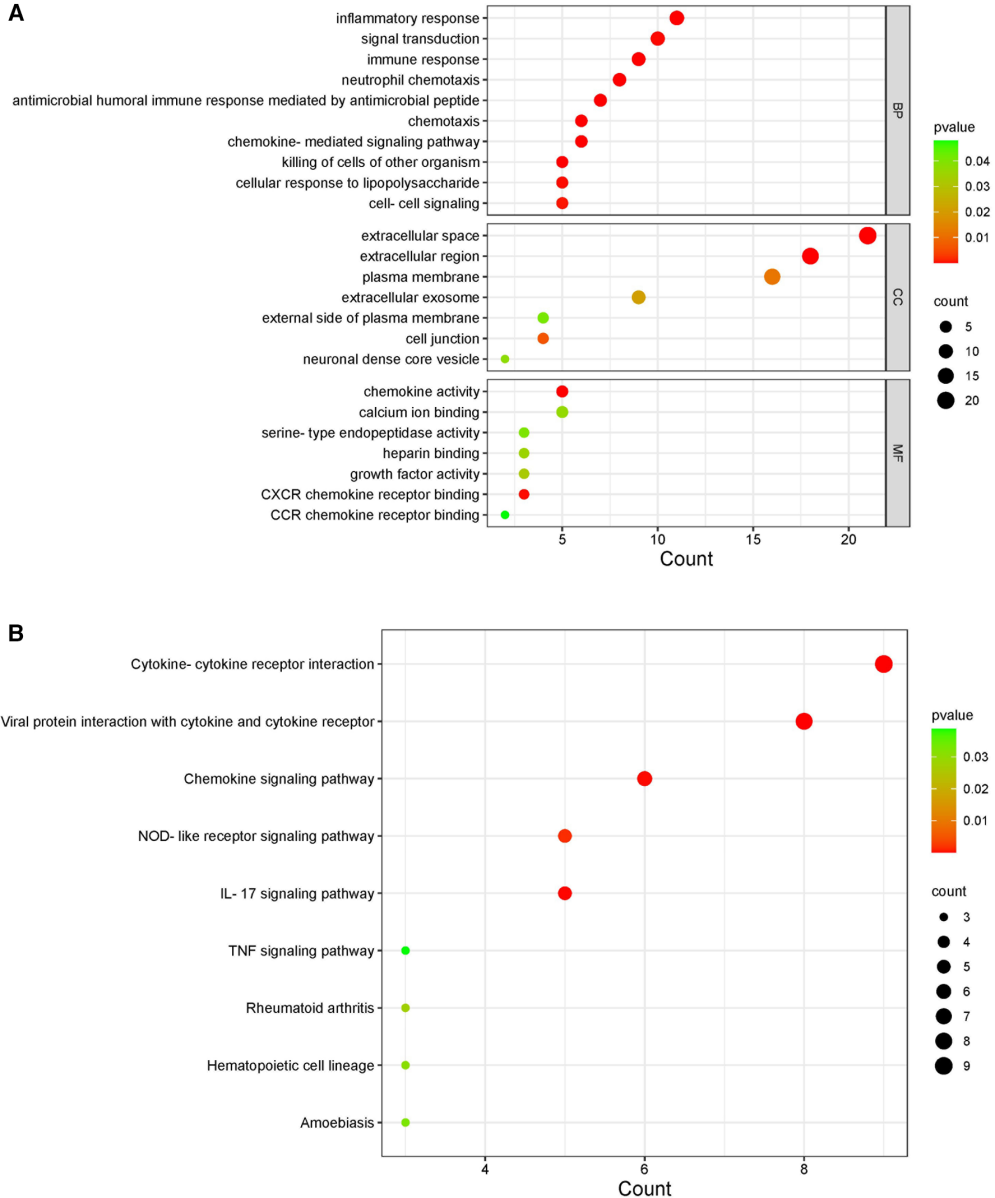
KEGG and GO diagrams of immune related genes. (**A**) GO analysis of the functional enrichment of immune-related genes. The dot size reflects the number of enriched genes, and the color indicates the significance of enrichment. (**B**) KEGG pathways of immune-related genes. The dot size reflects the number of enriched genes, and the color indicates the significance of enrichment.

By comparing the overlapping genes of HF and IBD with immune genes, 36 immune-related genes were obtained. BMP4 and SERPINA3 were deleted after screening in three samples, and 34 immune-related genes were ultimately obtained (Table 1). GO and KEGG analyses of immune-related genes. The GO analysis results of immune-related genes (Figure 3A) were mainly enriched in extracellular space, neutrophil chemotaxis, inflammatory response, extracellular region, antimicrobial humoral immune response mediated by antimicrobial peptide, chemokine-mediated signaling pathway, immune response, chemokine activity, chemotaxis, killing of cells of other organism signal transduction, and cellular response to lipopolysaccharide. The KEGG analysis results (Figure 3B) were mainly enriched in viral protein interaction with cytokine and cytokine receptor, cytokine-cytokine receptor interaction, IL-17 signaling pathway, chemokine signaling pathway and NOD-like receptor signaling pathway.

**Table 1:** Information on immune-related genes.

Immune related genes	HF Changes	IBD Changes	Immune related genes	HF Changes	IBD Changes
S100A9	↑	↑	PPBP	↑	↑
CXCR2	↑	↑	NR2F6	↑	↓
S100A11	↑	↑	PF4V1	↓	↑
IDO1	↑	↑	ICOS	↓	↑
PLAU	↓	↑	IL6	↓	↑
CCL11	↓	↑	RASGRP1	↑	↑
IFI30	↓	↑	EDN3	↑	↓
CCL2	↓	↑	NAMPT	↓	↑
TNFRSF12A	↑	↑	AVP	↑	↑
APLNR	↓	↑	CD19	↑	↑
PTGDR2	↑	↓	CD1B	↑	↑
IGLC1	↑	↑	LTF	↓	↑
CLEC11A	↑	↑	IL18RAP	↑	↑
SEMA4A	↑	↑	SEMG1	↓	↑
CXCL2	↑	↑	NLRX1	↑	↑
CELA1	↑	↓	SST	↑	↓
LTBP2	↑	↑	CDH1	↓	↓

### 3.3. Key gene screening

**Figure 4:**
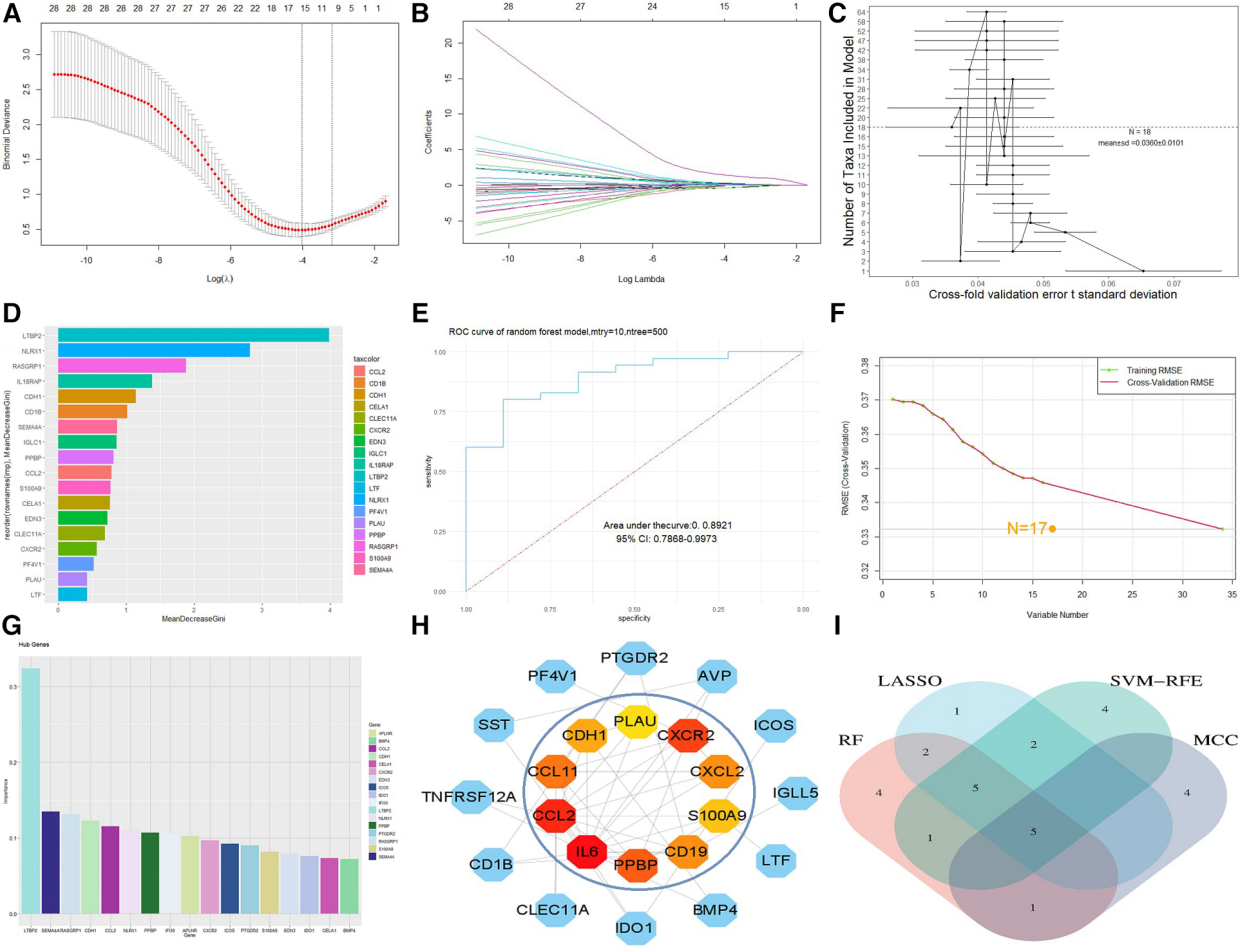
Machine model diagram and immune-related gene network. (**A**) Least absolute shrinkage and selection operator (LASSO) logistic regression algorithm to screen diagnostic markers. (**B**) Different colors represent different genes. (**C**) Cross-fold validation error *t* standard deviation in the random forest model. The red dashed line represents the number of genes with the smallest error. (**D**) Hub genes selected from the random forest model. The length of the bar chart represents the importance of the genes. (**E**) ROC curve of random forest. (**F**) Minimum root mean square error graph for feature gene selection in SVM. (**G**) The hub genes selected by SVM; the height of the bar graph represents the importance of the genes. (**H**) Top 10 hub genes of the immune-related gene network. (**I**) Overlapping genes of four algorithms.

For the LASSO algorithm, after ten cross validations, the best λ was 0.0180439 (Figures 4A,B). Therefore, due to the higher accuracy of the comparison, we selected the minimum standard for constructing LASSO classifiers and identified 15 characteristic genes, including IFI30, PPBP, S100A9, NLRX1, CCL2, CDH1, IGLC1, EDN3, SEMA4A, CXCR2, AVP, BMP4, LTBP2, APLNR, and RASGRP1. For the RF algorithm, the out of bag (OOB) error of the model was estimated to be 10.78%, and the under curve (AUC) was 0.8921 (Figure 4E). Eighteen relatively important characteristic genes (Figures 4C,D) that caused the least errors were identified, including BMP4, IL6, PF4V1, CELA1, CXCR2, CLEC11A, EDN3, S100A9, IGLC1, PPBP, CD1B, CCL2, SEMA4A, CDH1, IL18RAP, RASGRP1, NLRX1, and LTBP2. For the SVM-RFE algorithm, when the feature number was 17 (Figure 4F), the classifier had a minimum error, including LTBP2, IFI30, SEMA4A, CCL2, CDH1, APLNR, NLRX1, RASGRP1, CXCR2, PPBP, ICOS, PTGDR2, IDO1, S100A9, IL18RAP, IGLC1, and EDN3 (Figure 4G). After outputting PPI relationships in data format, visualization was achieved using Cytoscape 3.9.1 software. The cytoHubba plug-in was used to screen the top 10 key genes in DEG PPIs based on the MCC algorithm, including IL6, CCL2, CXCR2, PPBP, CCL11, CD19, CXCL2, S100A9, CDH1, and PLAU (Figure 4H). After comparison between sets, five characteristic genes, CCL2, CXCR2, PPBP, S100A9 and CDH1, shared by the LASSO, random forest, SVM-RFE, and MCC algorithms were finally identified (Figure 4I).

### 3.4. Verification of characteristic markers

**Figure 5:**
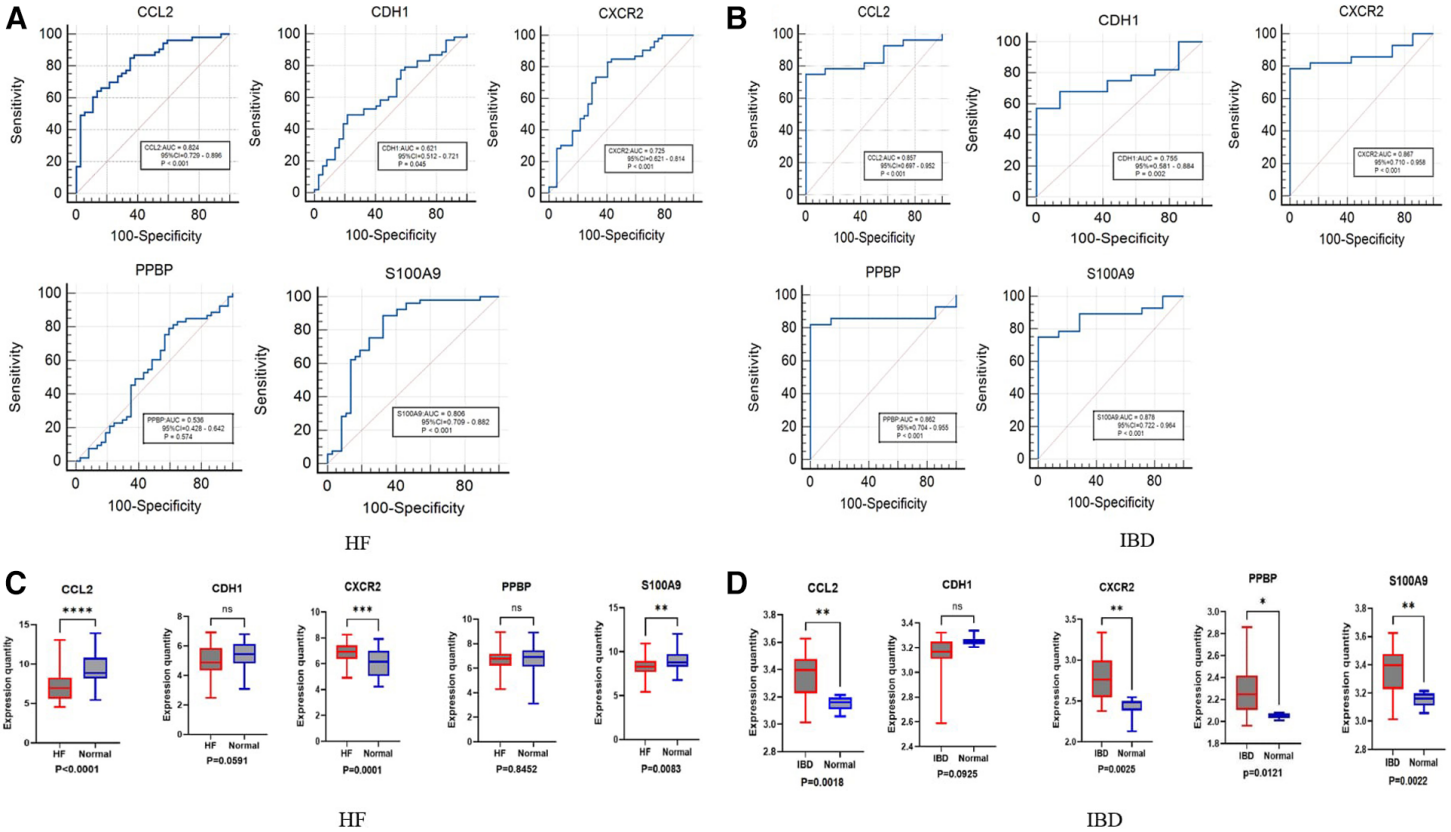
ROC and box diagram of biomarkers. (**A**) ROC diagnostic curve of key genes in HF samples. (**B**) ROC diagnostic curve of key genes in IBD samples. (**C**) Differential expression levels of five immune-related genes in the HF validation set: ****P < 0.0001, ***P < 0.001, **P < 0.01, *P < 0.05. (**D**) Differential expression levels in five immune-related genes in the inflammatory bowel disease validation set: ****P < 0.0001, ***P < 0.001, **P < 0.01, *P < 0.05.

We further evaluated the diagnostic values of CCL2, CDH1, CXCR2, PPBP and S100A9 through ROC curves (Figures 5A,B). CCL2, CXCR2, and S100A9 had high accuracy in HF (GSE1145) and IBD (GSE36807), AUC > 0.7. Statistical analysis was conducted using GraphPad Prism 9.5.1 software. Nonpaired sample *t* tests were performed on the expression levels of positive and normal samples, with a difference of *P* < 0.05 being statistically significant. These results are shown in (Figures 5C,D). Finally, the differences in the validation set were statistically significant. Combined with the key genes of the ROC curve as the screening results of this study, CCL2, CXCR2, and S100A9 were screened out. S100A9 and CXCR2 are upregulated in both diseases, while CCL2 is downregulated in HF and upregulated in IBD. It is believed that these key genes can be used as immune diagnostic markers for HF and IBD.

### 3.5. Immune cell infiltration

**Figure 6:**
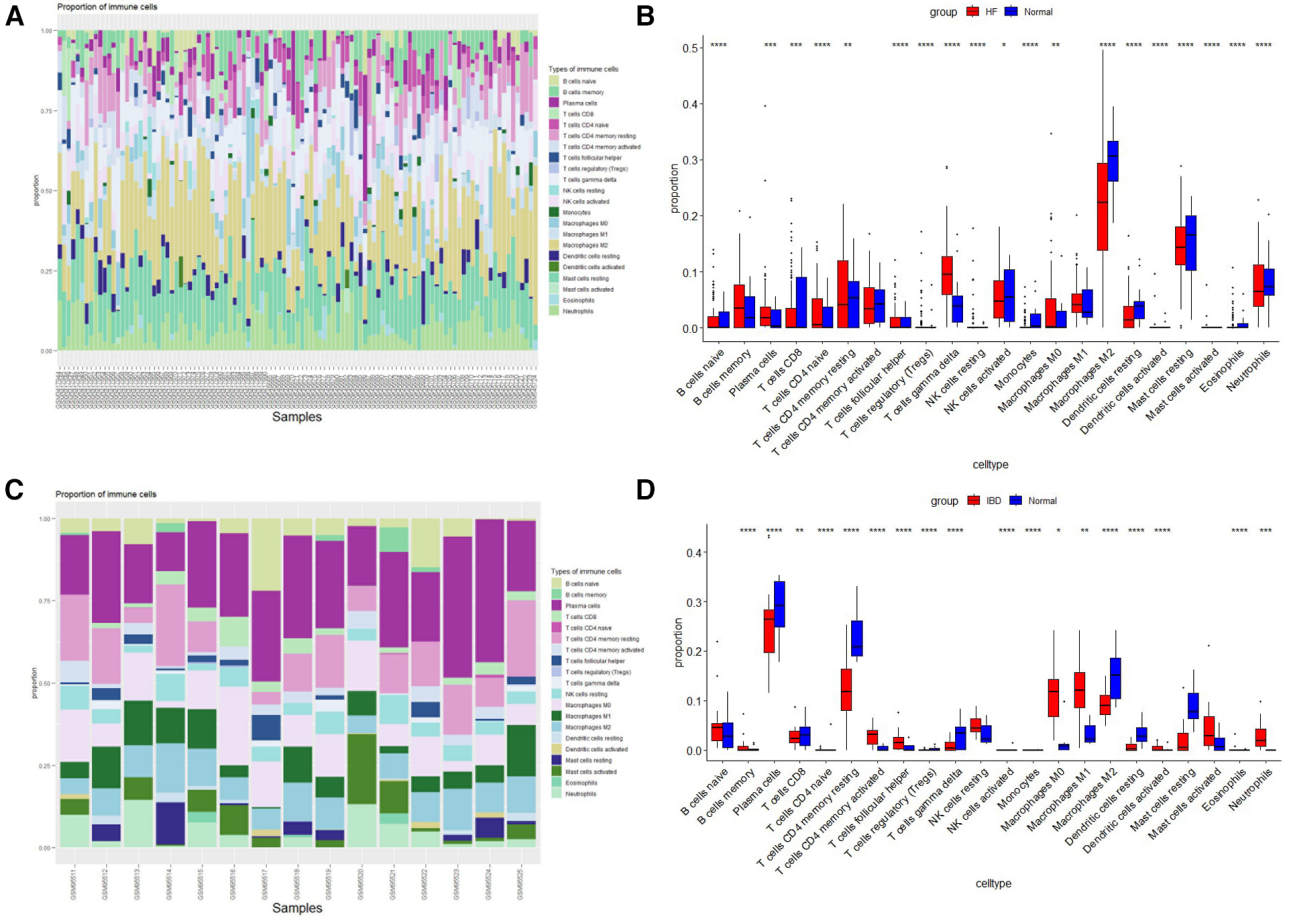
Immune cell infiltration. (**A**) Evaluation of infiltration of 22 types of immune cells in HF samples. (**B**) Violin diagram of the proportions of 22 types of immune cells in HF. (**C**) Evaluation of 22 types of immune cell infiltration in IBD samples. (**D**) Violin plot of the proportions of 22 types of immune cells in IBD.

The histogram shows the composition of 22 immune cells in the HF- and IBD-positive samples (Figures 6A,C). The colors of the histograms show the proportions of different immune cells in each sample, for a total of 1. The results indicate that in HF, resting memory CD4 *T*-cells, resting dendritic cells, memory B-cells, M2 macrophages, neutrophils, resting mast cells, gamma delta *T*-cells, and M0 macrophages are the main infiltrating immune cells. In IBD, M0 macrophages, M1 macrophages, M2 macrophages, neutrophils, resting NK cells, resting memory CD4 *T*-cells, CD8 *T*-cells, plasma cells and naïve B-cells are the main infiltrating immune cells. Both diseases present with resting memory CD4 *T*-cells, M0 macrophages, M2 macrophages, and neutrophils, indicating a correlation between the immune infiltration mechanisms of the two diseases. Then, the Wilcoxon test was used to determine significant differences in immune cell infiltration between the HF group and the control group, as well as between the IBD group and the control group, with *P* < 0.05 indicating a significant difference (Figures 6B,D). There were significant differences in 19 types of immune cells between the HF group and the control group. The HF group had significantly higher levels of gamma delta *T*-cells, neutrophils, resting memory CD4 *T*-cells, and M0 macrophages, and the control group had significantly higher levels of M2 macrophages, resting mast cells, and CD8 *T*-cells. There were significant differences in 18 types of immune cell infiltration between the IBD group and the control group. Compared with the control group, IBD patients had higher levels of activated memory CD4 *T*-cells, follicular helper *T*-cells, neutrophils, M0 macrophages, and M1 macrophages. In the control group, M2 macrophages, dendritic cell resetting, CD4 memory *T*-cell resetting, and plasma cells were significantly increased, and the overlapping results showed that neutrophils and M0 macrophages were higher in both diseases, while M2 macrophages were lower in both diseases.

### 3.6. Immune cell correlation with candidate biomarkers

**Figure 7:**
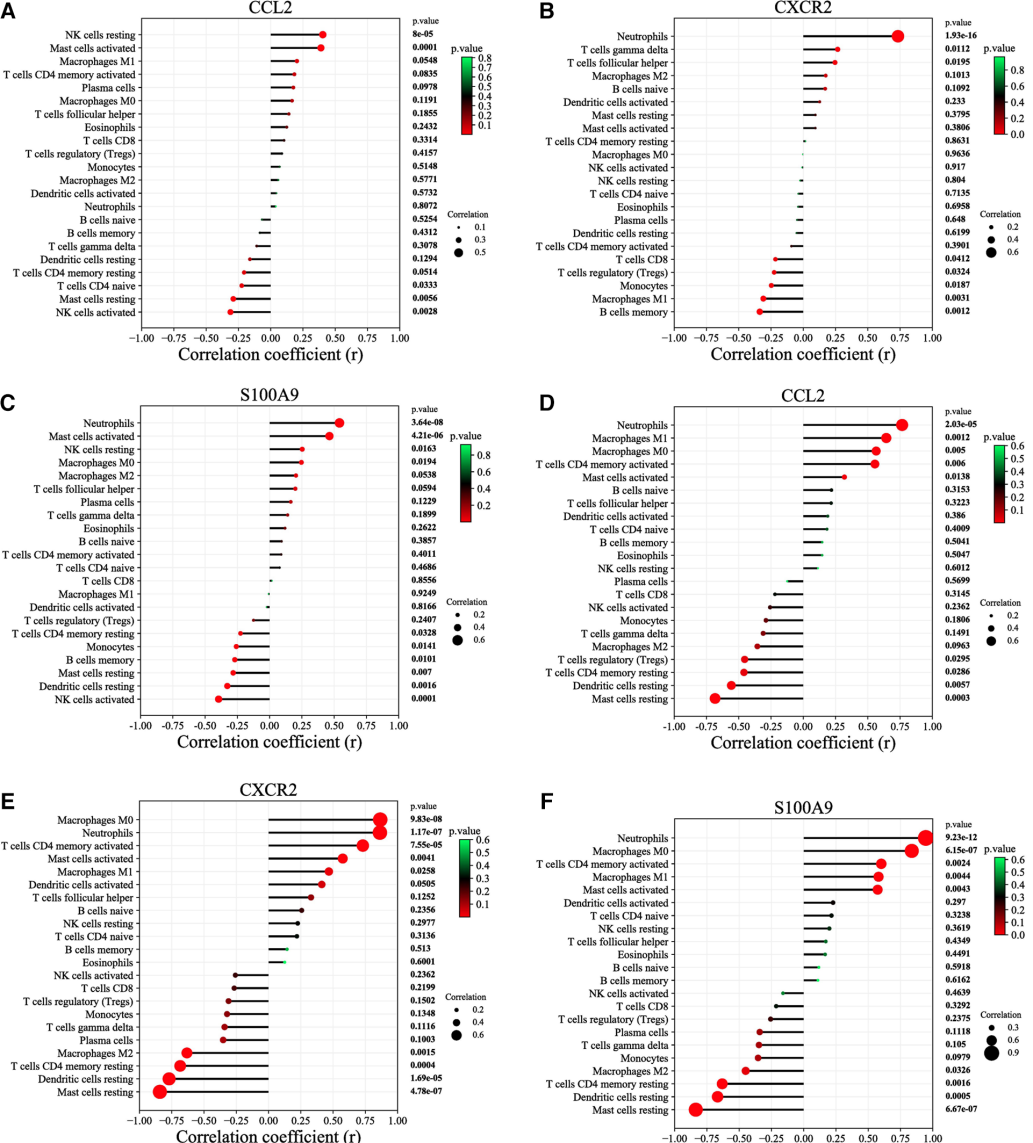
Correlation between diagnostic markers and infiltrating immune cells. (**A**) Correlation between CCL2 and infiltrating immune cells in HF. **(B**) Correlation between CXCR2 and infiltrating immune cells in HF. (**C**) Correlation between S100A9 and infiltrating immune cells in HF. (**D**) Correlation between CCL2 and infiltrating immune cells in IBD. (**E**) Correlation between CXCR2 and infiltrating immune cells in IBD. (**F**) Correlation between S100A9 and infiltrating immune cells in IBD. The size of the dots represents the strength of the correlation between genes and immune cells; the larger the dots, the stronger the correlation, and the smaller the dots, the weaker the correlation. The color of the dots represents the *P* value; the greener the color, the lower the *P* value; and the redder the color, the larger the *P* value. *P* < 0.05 was considered statistically significant.

According to the results of correlation analysis, in HF, CCL2 was positively correlated with NK cells resting (*r* = 0.4036, *p* = 8.00E-05) and Mast cells activated (*r* = 0.3893, *p* = 0.0001) (Figure 7A), CXCR2 was positively correlated with neutrophils (*r* = 0.7338, *p* = 1.93E-16) (Figure 7B), and S100A9 was positively correlated with activated neutrophils (*r* = 0.5412, *p* = 3.64E-8) and Mast cell activated (*r* = 0.4635, *p* = 4.21E-06) (Figure 7C). In IBD, CCL2 is positively correlated with neutrophils (*r* = 0.7661, *p* = 2.03E-05), macrophages M1(*r* = 0.6433, *p* = 0.0012), macrophages M0(*r* = 0.5649, *p* = 0.0050), *T* cell CD4 memory activated(*r* = 0.5552, *p* = 0.0060) and mast cells activated (*r* = 0.3188, p = 0.0138), CXCR2 is positively correlated with macrophages M0(*r* = 0.8653, *p* = 9.83E-08), neutrophils (*r* = 0.8630, *p* = 1.17E-07), *T* cells CD4 memory activated (*r* = 0.7305, *p* = 7.55E-05), mast cells activated (*r* = 0.5750, *p* = 0.0041), macrophages M1(*r* = 0.4674, *p* = 0.0258) and dendritic cells activated (*r* = 0.4124, *p* = 0.0505), S100A9 is positively correlated with neutrophils (*r* = 0.9462, *p* = 9.23E-12), macrophages M0(*r* = 0.8377, *p* = 6.15E-07), *T* cellsCD4 memory activated (*r* = 0.6013, *p* = 0.0024), macrophages M1(*r* = 0.5800, *p* = 0.0044), mast cells activated (*r* = 0.5730, *p* = 0.0043) (Figures 7D–F).

## 4. Discussion

HF is closely related to persistent chronic low-grade aseptic inflammation of the whole body and locally in the heart, as well as microvascular injury characterized by endothelial dysfunction, oxidative stress, myocardial remodeling, and fibrosis (25). IBD is characterized by recurrent episodes of gastrointestinal inflammation caused by an abnormal immune response to intestinal microflora (26). The relationship between the two diseases is still unclear, but immunity and inflammation play an important role in HF and in enteritis. Therefore, exploring the similarities in the infiltration of immune cells between the two diseases and investigation of immune-related biomarkers overlapping between the two diseases will undoubtedly have important clinical significance for the early recognition and intervention of these diseases.

The DEGs and immune genes of the two diseases were intersected to obtain overlapping immune-related genes, and the immune-related genes were analyzed by GO and KEGG. The GO analysis results were mainly related to neutrophil chemotaxis, inflammatory response, cathelicidin-mediated humoral immunity response, immune response, chemokine activity, and chemotaxis. KEGG analysis results were related to the interaction of viral protein with cytokines and cytokine receptor, cytokine-cytokine receptor interaction, IL-17 signaling pathway, chemokine signaling pathway, NOD-like receptor signaling pathway, rheumatoid arthritis, hematopoietic cell lineage, amebic disease, tumor necrosis factor signaling pathway, neuroactive ligand receptor interaction, and influenza A. Inflammatory cytokines such as tumor necrosis factor and various chemokines may stimulate the recruitment of proinflammatory white blood cells and promote myocardial injury, fibrosis remodeling and dysfunction, and promote the occurrence and development of IBD (19, 27). Cathelicidins (AMPs) are low-molecular weight proteins with broad-spectrum antibacterial and immunomodulatory activities that can fight against infectious bacteria (28) and play key roles in maintaining tolerance to intestinal microbiota and preventing intestinal infection (29). Gen Long Xue et al. speculate that IL-17 signaling bypasses NF-κB to inhibit the expression of SERCA2a and Cav1.2 in HF, thereby damaging the functional contraction and structural remodeling of myocardial cells and thus participating in the development of HF (30). NOD-like receptor signaling is related to myocardial remodeling and cardiomyocyte hypertrophy in HF (31, 32). Overexpression of IL-17 in IBD plays a key role in the disease mechanism (33), and IL-17 has become a therapeutic target for multiple sclerosis, psoriasis, psoriatic arthritis, ankylosing spondylitis, rheumatoid arthritis, and IBD (34, 35).

By combining bioinformatics analysis and machine learning, CCL2, CXCR2, S100A9, PPBP, and CDH1 were screened. After validation, CCL2, CXCR2, and S100A9 were used as immune-related gene markers for the two diseases. CCL2, also known as monocyte chemoattractant protein-1 (MCP-1), comes from the CC chemokine family (36) and is a small molecule (5–20 kDa) basic heparin binding protein with 20%–70% homology in the amino acid sequence, characterized by the conserved position of four cysteine residues (37). CCL2 is produced by many cell types, including endothelial cells, fibroblasts, epithelial cells, smooth muscle cells, mesangial cells, astrocytes, monocytes and microglia (38, 39). CCL2 can promote cardiac decompensation (40), and upregulation in stressed and injured cardiomyocytes (41). TFRC in cardiomyocytes recruits and activates macrophages by secreting CCL2 to induce myocardial hypertrophy and promote HF development (42). Daniela Impellizzeri et al. found that pro-inflammatory mediator CCL2 was significantly upregulated in inflammation of the colon and is associated with increased disease activity (43). CXCR2 is a chemokine receptor classified as a G protein-coupled receptor (GPCR) belonging to the CXCR family, and it is the receptor for CXC motif chemokine ligand 8 (CXCL8)/Interleukin 8 (IL-8) (44). It is expressed in various cell types, such as neutrophils, monocytes, eosinophils, endothelial cells, mast cells and oligodendrocytes (45). Yun-Long Zhang et al. found that CXCR2 levels are elevated in mice injected with angiotensin II and heart failure patients. Inhibiting CXCR2 can inhibit the progression of established cardiac contractile dysfunction, hypertrophy, and fibrosis, reducing myocardial inflammation and oxidative stress. Thus inhibiting the progression of cardiac hypertrophy remodeling, cardiac remodeling, and dysfunction (46), which play a key role in the pathogenesis of HF. Lei Wang et al. found that CXCR2-mediated signaling pathways exacerbate cardiac remodeling (47), and inhibiting CXCR2 can inhibit the progression of established cardiac systolic dysfunction, hypertrophy, and fibrosis (46). In IBD, Blocking CXCR2 signaling could be a potential therapeutic target for the prevention of IBD (48). CXCR2 has become a therapeutic target for many inflammatory diseases, such as chronic obstructive pulmonary disease (COPD), allergic asthma, gram-negative sepsis, IBD, lung injury, auto-immune diseases and cancer (45, 49). S100A9, also known as myeloid-associated protein 14 or calpain B, belongs to the calcium-binding protein S100 family and is mainly expressed in neutrophils. In addition, under the influence of various chronic inflammatory factors, it can also be expressed on vascular endothelial cells and mature macrophages (50). S100A9 has a pleiotropic effect on the cardiovascular system. Xuan Wei et al. found that hypertrophic myocardial precursors can reduce myocardial fibrosis and cardiac muscle cell hypertrophy by upregulating S100A9 (51). MARINKOVIC G et al. found that short-term S100A9 blockade improves cardiac function after permanent myocardial ischemia in mice (52). The study found that S100A8 and S100A9 mRNA were differentially expressed in blood leukocytes of patients with IBD compared to healthy controls and in active compared to quiescent disease. S100A9 could therefore be used as a potential biomarker of IBD (53). The above findings further confirm the great potential of CCL2, CXCR2 and S100A9 as immune-related biomarkers of these two diseases.

CIBERSORT was applied to evaluate the immune infiltration process in HF and IBD. *T* cell CD4 memory resting, macrophages M0, macrophages M2, neutrophils are present in both diseases, with levels of neutrophils and Macrophages M0 being higher in both diseases and levels of Macrophages M2 being lower in both diseases. These immune cells may be related to the occurrence and development of both diseases. Neutrophils are the main phagocytes in the circulating blood and reach the inflammatory site in a cascade-like manner, leading to activation of specific effector functions (54). The increase in neutrophils and sustained activation of neutrophils are the main factors determining the overactivated inflammation of acute HF and the long-term outcomes of chronic HF (55). Macrophages are the main immune cell group in the resting heart tissue and are present around interstitial and endothelial cells (56). The M2 phenotype is involved in tissue repair and immune tolerance and play a role in maintaining organs and soft tissues and regulating the immune balance (57), exhibits cardioprotective effects in HF (58). In the gastrointestinal tract, macrophages are considered to play an important role in maintaining the stability of the internal intestinal environment and are also the key sentries of the intestinal immune system (59). There is evidence to suggest a causal relationship between the deficiency of intestinal inflammation regression and changes in monocyte macrophage differentiation in IBD patients (60).

Regarding correlation between immune-related biomarkers and immune cells, CCL2 was positively correlated with mast cells activated and negatively correlated with mast cells resting in both HF and IBD. CXCR2 is positively correlated with neutrophils, and S100A9 is positively correlated with neutrophils and mast cells activated and negatively correlated with Dendritic cells resetting and Mast cells resetting. In the intestinal mucosa, CCL2 attracts mast cells, and MC activation on the mucosal surface mediates a severe inflammatory response (61). In HF, mast cells are activated to induce the production of CCL2 (62). CXCR2 and S100A9 are neutrophil-related biomarkers and potential therapeutic targets in ulcerative colitis (63). AVERILL MM et al. found that S100A9 can change the phenotypic status of neutrophils, macrophages and dendritic cells to varying degrees and is related to inflammation, IBD, obesity, and cardiovascular disease (48, 64, 65). These results reveal the interaction between genes and immune cells.

This paper used machine learning and bioinformatics methods to explore the immune-related mechanisms, immune infiltrating cells and immune-related biomarkers of HF and IBD. In addition to researching the mechanism of immune-related genes using GO and KEGG, we used RF, Lasso regression, and the SVM-RFE algorithm to identify characteristic genes, and CIBERSORT was used to complete immune cell infiltration for the two diseases. However, this study has many shortcomings. First, there is a lack of validation of large-sample clinical and basic trial results. Second, there are too few high-quality articles on the relationship between enteric diseases and HF.

## 5. Conclusion

This study screened C-C motif chemokine 2(CCL2), C-X-C chemokine receptor type 2(CXCR2), and Protein S100-A9(S100A9) as immune-related diagnostic markers for HF and IBD. Neutrophils, M0 macrophages, and M1 macrophages may be involved in the occurrence and development of both diseases. In addition, CCL2 is positively correlated with mast cells activated in both HF and IBD, CXCR2 is positively correlated with neutrophils, and S100A9 is positively correlated with neutrophils and mast cells activated. In summary, these immune cells and immune-related diagnostic markers may have a significant impact on the development of HF and IBD. The study of immune cells between IBD and HF and the identification of relevant diagnostic markers may determine the targets of immunotherapy, providing ideas for the immunomodulatory treatment of these two diseases in clinical practice.

## Data Availability

The original contributions presented in the study are included in the article/Supplementary Material, further inquiries can be directed to the corresponding author.

## References

[1] Heidenreich PA Bozkurt B Aguilar D Allen LA Byun JJ Colvin MM 2022 AHA/ACC/HFSA guideline for the management of heart failure: executive summary: a report of the American College of Cardiology/American Heart Association joint committee on clinical practice guidelines. Circulation. (2022) 145:e876–94. 10.1161/CIR.0000000000001062 35363500

[2] Savarese G Becher PM Lund LH Seferovic P Rosano GMC Coats AJS. Global burden of heart failure: a comprehensive and updated review of epidemiology. Cardiovasc Res. (2023) 118:3272–87. 10.1093/cvr/cvac013 35150240

[3] Dick SA Epelman S. Chronic heart failure and inflammation: what do we really know? Circ Res. (2016) 119:159–76. 10.1161/CIRCRESAHA.116.308030 27340274

[4] Triposkiadis F Xanthopoulos A Parissis J Butler J Farmakis D. Pathogenesis of chronic heart failure: cardiovascular aging, risk factors, comorbidities, and disease modifiers. Heart Fail Rev. (2022) 27:337–44. 10.1007/s10741-020-09987-z 32524327

[5] Redfield MM Borlaug BA. Heart failure with preserved ejection fraction: a review. JAMA. (2023) 329:827–38. 10.1001/jama.2023.2020 36917048

[6] La Franca E Manno G Ajello L Di Gesaro G Mina C Visconti C Physiopathology and diagnosis of congestive heart failure: consolidated certainties and new perspectives. Curr Probl Cardiol. (2021) 46:100691. 10.1016/j.cpcardiol.2020.100691 33012532

[7] Schefold JC Filippatos G Hasenfuss G Anker SD von Haehling S. Heart failure and kidney dysfunction: epidemiology, mechanisms and management. Nat Rev Nephrol. (2016) 12:610–23. 10.1038/nrneph.2016.113 27573728

[8] Zhang Y Bauersachs J Langer HF. Immune mechanisms in heart failure. Eur J Heart Fail. (2017) 19:1379–89. 10.1002/ejhf.942 28891154

[9] Lu Y Xia N Cheng X. Regulatory T cells in chronic heart failure. Front Immunol. (2021) 12:732794. 10.3389/fimmu.2021.732794 34630414 PMC8493934

[10] Porter RJ Kalla R Ho GT. Ulcerative colitis: recent advances in the understanding of disease pathogenesis. F1000Res. (2020) 9:294. 10.12688/f1000research.20805.1 PMC7194476 32399194

[11] Kofla-Dlubacz A Pytrus T Akutko K Sputa-Grzegrzolka P Piotrowska A Dziegiel P. Etiology of IBD-is it still a mystery? Int J Mol Sci. (2022) 23:12445. 10.3390/ijms232012445 36293300 PMC9604112

[12] Agrawal M Jess T. Implications of the changing epidemiology of inflammatory bowel disease in a changing world. United European Gastroenterol J. (2022) 10:1113–20. 10.1002/ueg2.12317 36251359 PMC9752308

[13] Aniwan S Santiago P Loftus EV Jr Park SH. The epidemiology of inflammatory bowel disease in Asia and Asian immigrants to western countries. United European Gastroenterol J. (2022) 10:1063–76. 10.1002/ueg2.12350 36479863 PMC9752270

[14] Ramos GP Papadakis KA. Mechanisms of disease: inflammatory bowel diseases. Mayo Clin Proc. (2019) 94:155–65. 10.1016/j.mayocp.2018.09.013 30611442 PMC6386158

[15] Saez A Gomez-Bris R Herrero-Fernandez B Mingorance C Rius C Gonzalez-Granado JM. Innate lymphoid cells in intestinal homeostasis and inflammatory bowel disease. Int J Mol Sci. (2021) 22:7618. 10.3390/ijms22147618 34299236 PMC8307624

[16] Malik TF Aurelio DM. Extraintestinal manifestations of inflammatory bowel disease. StatPearls. Treasure Island (FL) ineligible companies. Disclosure: Danilo Aurelio declares no relevant financial relationships with ineligible companies. (2023). Bookshelf ID: NBK568797.

[17] Kumar A Lukin DJ. Incident heart failure is a predictor of adverse outcomes in inflammatory bowel disease. Eur J Gastroenterol Hepatol. (2020) 32:205–15. 10.1097/MEG.0000000000001648 31851091

[18] Chen B Collen LV Mowat C Isaacs KL Singh S Kane SV Inflammatory bowel disease and cardiovascular diseases. Am J Med. (2022) 135:1453–60. 10.1016/j.amjmed.2022.08.012 36058305

[19] Saez A Herrero-Fernandez B Gomez-Bris R Sanchez-Martinez H Gonzalez-Granado JM. Pathophysiology of inflammatory bowel disease: innate immune system. Int J Mol Sci. (2023) 24:1526. 10.3390/ijms24021526 36675038 PMC9863490

[20] Martini E Kunderfranco P Peano C Carullo P Cremonesi M Schorn T Single-cell sequencing of mouse heart immune infiltrate in pressure overload-driven heart failure reveals extent of immune activation. Circulation. (2019) 140:2089–107. 10.1161/CIRCULATIONAHA.119.041694 31661975

[21] Auslander N Gussow AB Koonin EV. Incorporating machine learning into established bioinformatics frameworks. Int J Mol Sci. (2021) 22:2903. 10.3390/ijms22062903 33809353 PMC8000113

[22] Xie Y Shi H Han B. Bioinformatic analysis of underlying mechanisms of kawasaki disease via weighted gene correlation network analysis (WGCNA) and the least absolute shrinkage and selection operator method (LASSO) regression model. BMC Pediatr. (2023) 23:90. 10.1186/s12887-023-03896-4 36829193 PMC9951419

[23] Strobl C Boulesteix AL Zeileis A Hothorn T. Bias in random forest variable importance measures: illustrations, sources and a solution. BMC Bioinformatics. (2007) 8:25. 10.1186/1471-2105-8-25 17254353 PMC1796903

[24] Sanz H Valim C Vegas E Oller JM Reverter F. SVM-RFE: selection and visualization of the most relevant features through non-linear kernels. BMC Bioinformatics. (2018) 19:432. 10.1186/s12859-018-2451-4 30453885 PMC6245920

[25] Ling S Xu JW. NETosis as a pathogenic factor for heart failure. Oxid Med Cell Longev. (2021) 2021:6687096. 10.1155/2021/6687096 33680285 PMC7929675

[26] McDowell C Farooq U Haseeb M. Inflammatory bowel disease. StatPearls. Treasure Island (FL) with ineligible companies. Disclosure: Umer Farooq declares no relevant financial relationships with ineligible companies. Disclosure: Muhammad Haseeb declares no relevant financial relationships with ineligible companies. (2023). Bookshelf ID: NBK470312.

[27] Hanna A Frangogiannis NG. Inflammatory cytokines and chemokines as therapeutic targets in heart failure. Cardiovasc Drugs Ther. (2020) 34:849–63. 10.1007/s10557-020-07071-0 32902739 PMC7479403

[28] Boparai JK Sharma PK. Mini review on antimicrobial peptides, sources, mechanism and recent applications. Protein Pept Lett. (2020) 27:4–16. 10.2174/18755305MTAwENDE80 31438824 PMC6978648

[29] Gubatan J Holman DR Puntasecca CJ Polevoi D Rubin SJ Rogalla S. Antimicrobial peptides and the gut microbiome in inflammatory bowel disease. World J Gastroenterol. (2021) 27:7402–22. 10.3748/wjg.v27.i43.7402 34887639 PMC8613745

[30] Xue GL Li DS Wang ZY Liu Y Yang JM Li CZ Interleukin-17 upregulation participates in the pathogenesis of heart failure in mice via NF-kappaB-dependent suppression of SERCA2a and Cav1.2 expression. Acta Pharmacol Sin. (2021) 42:1780–9. 10.1038/s41401-020-00580-6 33589793 PMC8563866

[31] Wu YX Xu RY Jiang L Chen XY Xiao XJ. MicroRNA-30a-5p promotes chronic heart failure in rats by targeting sirtuin-1 to activate the nuclear factor-kappaB/NOD-like receptor 3 signaling pathway. Cardiovasc Drugs Ther. (2022). 10.1007/s10557-021-07304-w 35488974

[32] Tang X Pan L Zhao S Dai F Chao M Jiang H SNO-MLP (S-Nitrosylation of muscle LIM protein) facilitates myocardial hypertrophy through TLR3 (toll-like receptor 3)-mediated RIP3 (receptor-interacting protein kinase 3) and NLRP3 (NOD-like receptor pyrin domain containing 3) inflammasome activation. Circulation. (2020) 141:984–1000. 10.1161/CIRCULATIONAHA.119.042336 31902237

[33] G JM de Morales R Puig L Dauden E Canete JD Pablos JL Critical role of interleukin (IL)-17 in inflammatory and immune disorders: an updated review of the evidence focusing in controversies. Autoimmun Rev. (2020) 19:102429. 10.1016/j.autrev.2019.102429 31734402

[34] Fauny M Moulin D D'Amico F Netter P Petitpain N Arnone D Paradoxical gastrointestinal effects of interleukin-17 blockers. Ann Rheum Dis. (2020) 79:1132–8. 10.1136/annrheumdis-2020-217927 32719044

[35] Kumar R Theiss AL Venuprasad K. RORgammat protein modifications and IL-17-mediated inflammation. Trends Immunol. (2021) 42:1037–50. 10.1016/j.it.2021.09.005 34635393 PMC8556362

[36] Singh S Anshita D Ravichandiran V. MCP-1: function, regulation, and involvement in disease. Int Immunopharmacol. (2021) 101:107598. 10.1016/j.intimp.2021.107598 34233864 PMC8135227

[37] Van Coillie E Van Damme J Opdenakker G. The MCP/eotaxin subfamily of CC chemokines. Cytokine Growth Factor Rev. (1999) 10:61–86. 10.1016/S1359-6101(99)00005-2 10379912

[38] Standiford TJ Kunkel SL Phan SH Rollins BJ Strieter RM. Alveolar macrophage-derived cytokines induce monocyte chemoattractant protein-1 expression from human pulmonary type II-like epithelial cells. J Biol Chem. (1991) 266:9912–8. 10.1016/S0021-9258(18)92905-4 2033076

[39] Deshmane SL Kremlev S Amini S Sawaya BE. Monocyte chemoattractant protein-1 (MCP-1): an overview. J Interferon Cytokine Res. (2009) 29:313–26. 10.1089/jir.2008.0027 19441883 PMC2755091

[40] Zhang H Yang K Chen F Liu Q Ni J Cao W Role of the CCL2-CCR2 axis in cardiovascular disease: pathogenesis and clinical implications. Front Immunol. (2022) 13:975367. 10.3389/fimmu.2022.975367 36110847 PMC9470149

[41] Li R Frangogiannis NG. Chemokines in cardiac fibrosis. Curr Opin Physiol. (2021) 19:80–91. 10.1016/j.cophys.2020.10.004 33195890 PMC7665080

[42] Pan Y Yang J Dai J Xu X Zhou X Mao W. TFRC in cardiomyocytes promotes macrophage infiltration and activation during the process of heart failure through regulating Ccl2 expression mediated by hypoxia inducible factor-1alpha. Immun Inflamm Dis. (2023) 11:e835. 10.1002/iid3.835 37647427 PMC10461419

[43] Impellizzeri D Fusco R Genovese T Cordaro M D'Amico R Trovato Salinaro A Coriolus versicolor downregulates TLR4/NF-kappaB signaling cascade in dinitrobenzenesulfonic acid-treated mice: a possible mechanism for the anti-colitis effect. Antioxidants (Basel). (2022) 11:406. 10.3390/antiox11020406 35204289 PMC8869697

[44] Korbecki J Kupnicka P Chlubek M Goracy J Gutowska I Baranowska-Bosiacka I. CXCR2 receptor: regulation of expression, signal transduction, and involvement in cancer. Int J Mol Sci. (2022) 23:2168. 10.3390/ijms23042168 35216283 PMC8878198

[45] Cheng Y Ma XL Wei YQ Wei XW. Potential roles and targeted therapy of the CXCLs/CXCR2 axis in cancer and inflammatory diseases. Biochim Biophys Acta Rev Cancer. (2019) 1871:289–312. 10.1016/j.bbcan.2019.01.005 30703432

[46] Zhang YL Geng C Yang J Fang J Yan X Li PB Chronic inhibition of chemokine receptor CXCR2 attenuates cardiac remodeling and dysfunction in spontaneously hypertensive rats. Biochim Biophys Acta Mol Basis Dis. (2019) 1865:165551. 10.1016/j.bbadis.2019.165551 31494226

[47] Wang L Zhang YL Lin QY Liu Y Guan XM Ma XL CXCL1-CXCR2 axis mediates angiotensin II-induced cardiac hypertrophy and remodelling through regulation of monocyte infiltration. Eur Heart J. (2018) 39:1818–31. 10.1093/eurheartj/ehy085 29514257

[48] Yao Z Zhang B Niu G Yan Z Tong X Zou Y Neutrophil infiltration characterized by upregulation of S100A8, S100A9, S100A12 and CXCR2 is associated with the co-occurrence of crohn’s disease and peripheral artery disease. Front Immunol. (2022) 13:896645. 10.3389/fimmu.2022.896645 35795659 PMC9251382

[49] Shi X Wan Y Wang N Xiang J Wang T Yang X Selection of a picomolar antibody that targets CXCR2-mediated neutrophil activation and alleviates EAE symptoms. Nat Commun. (2021) 12:2547. 10.1038/s41467-021-22810-z 33953162 PMC8100106

[50] Du F Ding Z Ronnow CF Rahman M Schiopu A Thorlacius H. S100a9 induces reactive oxygen species-dependent formation of neutrophil extracellular traps in abdominal sepsis. Exp Cell Res. (2022) 421:113405. 10.1016/j.yexcr.2022.113405 36328195

[51] Wei X Wu B Zhao J Zeng Z Xuan W Cao S Myocardial hypertrophic preconditioning attenuates cardiomyocyte hypertrophy and slows progression to heart failure through upregulation of S100A8/A9. Circulation. (2015) 131:1506–17. discussion 17. 10.1161/CIRCULATIONAHA.114.013789 25820336 PMC4415966

[52] Marinkovic G Grauen Larsen H Yndigegn T Szabo IA Mares RG de Camp L Inhibition of pro-inflammatory myeloid cell responses by short-term S100A9 blockade improves cardiac function after myocardial infarction. Eur Heart J. (2019) 40:2713–23. 10.1093/eurheartj/ehz461 31292614

[53] Azramezani Kopi T Amini Kadijani A Parsian H Shahrokh S Asadzadeh Aghdaei H Mirzaei A The value of mRNA expression of S100A8 and S100A9 as blood-based biomarkers of inflammatory bowel disease. Arab J Gastroenterol. (2019) 20:135–40. 10.1016/j.ajg.2019.07.002 31563476

[54] Margraf A Lowell CA Zarbock A. Neutrophils in acute inflammation: current concepts and translational implications. Blood. (2022) 139(14):2130–44. 10.1182/blood.2021012295 34624098 PMC9728535

[55] Kain V Halade GV. Role of neutrophils in ischemic heart failure. Pharmacol Ther. (2020) 205:107424. 10.1016/j.pharmthera.2019.107424 31629005 PMC6981275

[56] Lafuse WP Wozniak DJ Rajaram MVS. Role of cardiac macrophages on cardiac inflammation, fibrosis and tissue repair. Cells. (2020) 10:51. 10.3390/cells10010051 33396359 PMC7824389

[57] Kadomoto S Izumi K Mizokami A. Macrophage polarity and disease control. Int J Mol Sci. (2021) 23(1):144. 10.3390/ijms23010144 35008577 PMC8745226

[58] Liu Y Wu M Zhong C Xu B Kang L. M2-like macrophages transplantation protects against the doxorubicin-induced heart failure via mitochondrial transfer. Biomater Res. (2022) 26:14. 10.1186/s40824-022-00260-y 35410296 PMC8996664

[59] Bain CC Schridde A. Origin, differentiation, and function of intestinal macrophages. Front Immunol. (2018) 9:2733. 10.3389/fimmu.2018.02733 30538701 PMC6277706

[60] Na YR Stakenborg M Seok SH Matteoli G. Macrophages in intestinal inflammation and resolution: a potential therapeutic target in IBD. Nat Rev Gastroenterol Hepatol. (2019) 16:531–43. 10.1038/s41575-019-0172-4 31312042

[61] Conti P Caraffa A Ronconi G Kritas SK Mastrangelo F Tettamanti L Impact of mast cells in mucosal immunity of intestinal inflammation: inhibitory effect of IL-37. Eur J Pharmacol. (2018) 818:294–9. 10.1016/j.ejphar.2017.09.044 28970014

[62] Kinoshita M Okada M Hara M Furukawa Y Matsumori A. Mast cell tryptase in mast cell granules enhances MCP-1 and interleukin-8 production in human endothelial cells. Arterioscler Thromb Vasc Biol. (2005) 25:1858–63. 10.1161/01.ATV.0000174797.71708.97 15976326

[63] Muthas D Reznichenko A Balendran CA Bottcher G Clausen IG Karrman Mardh C Neutrophils in ulcerative colitis: a review of selected biomarkers and their potential therapeutic implications. Scand J Gastroenterol. (2017) 52:125–35. 10.1080/00365521.2016.1235224 27610713

[64] Mihaila AC Ciortan L Macarie RD Vadana M Cecoltan S Preda MB Transcriptional profiling and functional analysis of N1/N2 neutrophils reveal an immunomodulatory effect of S100A9-blockade on the pro-inflammatory N1 subpopulation. Front Immunol. (2021) 12:708770. 10.3389/fimmu.2021.708770 34447377 PMC8384118

[65] Franz S Ertel A Engel KM Simon JC Saalbach A. Overexpression of S100A9 in obesity impairs macrophage differentiation via TLR4-NFkB-signaling worsening inflammation and wound healing. Theranostics. (2022) 12(4):1659–82. 10.7150/thno.67174 35198063 PMC8825590

